# Family socioeconomic position and changes in planned health care for children with chronic diseases during the COVID-19 pandemic in Italy

**DOI:** 10.3389/fpubh.2023.1174118

**Published:** 2023-07-13

**Authors:** Giovenale Moirano, Costanza Pizzi, Franca Rusconi, Milena Maule, Lorenzo Richiardi, Maja Popovic

**Affiliations:** ^1^Cancer Epidemiology Unit and CPO-Piemonte, Department of Medical Sciences, University of Torino, Torino, Italy; ^2^Department of Public Health and Paediatrics, Post Graduate School of Medical Statistics, University of Torino, Torino, Italy; ^3^Department of Mother and Child Health, Azienda USL Toscana Nord Ovest, Pisa, Italy

**Keywords:** COVID-19 pandemic, planned health care, pediatric diseases, health inequalities, life-course epidemiology

## Abstract

**Introduction:**

In this study, we aimed at evaluating whether, during the COVID-19 pandemic, children affected by chronic diseases were impacted by the deferral of planned healthcare caused by the restriction measures.

**Design:**

This study was conducted using data from the Italian NINFEA birth cohort, which include children born between 2005 and 2016. Women who completed the 4-year NINFEA follow-up questionnaire before November 2020 (*N* = 5,307) were invited to complete a questionnaire targeted at evaluating the impacts of the pandemic on their children's health. The questionnaire asked mothers to report whether their children had a chronic disease or condition that required one or more regular health checks by a doctor in 2019 (used as a reference period) and whether the children had problems getting routine health checks after March 2020.

**Results:**

We obtained information on 3,721 children. Out of 353 children with a chronic disease that required at least one medical visit in 2019, 130 (36.8%) experienced problems during the pandemic. Lower family income was associated with a higher risk of experiencing health access problems. We observed that children living in families at lower income tertiles had more chance of experiencing healthcare access problems than children living in families at the highest income tertiles (prevalence rate ratio for a tertile decrease in family income: 1.22; 95% CIs: 1.02–1.49).

**Conclusion:**

Our study underlines that the COVID-19 pandemic may have caused healthcare access problems for children with prevalent chronic diseases, especially among those living in households with a low socioeconomic position.

## Introduction

Starting from March 2020, after the exponential increase in the number of COVID-19 cases, the Italian government imposed massive restrictions to reduce the spread of the disease (1). The healthcare sector was affected by the partial or complete interruption of diagnostic workflows causing lower hospital admissions in adults (2). In addition, changes in health-seeking behavior and the perception of hospitals as high-risk areas of SARS-CoV-2 transmission have also likely contributed to healthcare access reduction (3). These restrictions, with varying intensities, remained after the main lockdown period (8 March 2020–3 May 2020) throughout 2020 and for part of 2021. All these changes are likely to have an impact on children's health by decreasing the rates of pediatric emergency department visits, limiting physical activity, affecting sociality, worsening mental health, and interrupting or postponing pediatric outpatient appointments (4, 5). Children affected by chronic diseases have multiple and complex needs and were likely affected by changes in the delivery of care. However, few data are available on this topic or are limited to specific chronic health conditions (6). We aimed to evaluate whether, in the population-based Italian Nascita e Infanzia: gli Effetti dell'Ambiente (NINFEA) birth cohort, children with chronic diseases diagnosed before 2020 experienced any problems in making regular health checks between March 2020 and the beginning of 2021 due to the COVID-19 pandemic. In addition, since access to health services and adequate care has been previously shown to be influenced by the burden of comorbidities and socioeconomic position (SEP), we evaluated whether having experienced problems between March 2020 and the beginning of 2021 was associated with these factors.

## Methods

The NINFEA study is an Italian internet-based birth cohort (www.progettoninfea.it). Between 2005 and 2016, ~7,500 pregnant women were recruited by filling out a baseline questionnaire. Further details are discussed elsewhere (7). Briefly, mothers were invited to fill out follow-up questionnaires 6 and 18 months after delivery and when the children turn 4, 7, 10, 13, and 16 years of age. The NINFEA study was approved by the Ethical Committee of the San Giovanni Battista Hospital and CTO/CRF/Maria Adelaide Hospital of Turin (project number 45). Maternal written consent was obtained at enrolment and for each follow-up questionnaire. Women who completed the 4-year NINFEA follow-up questionnaire before November 2020 (*N* = 5,307) were invited to complete an *ad hoc* questionnaire that was released on 15 November 2020 and ended on 1 June 2021. The complete version of the questionnaire can be found online (www.progettoninfea.it/tour). Overall, the questionnaire aimed to evaluate the occurrence of SARS-CoV-2 infection and transmission among family members, potential impacts of the pandemic on family socioeconomic conditions (e.g., job interruption/loss experienced by parents, and changes in the family income), children and mothers mental health status, as well as problems in accessing healthcare system by children with chronic diseases. With regards to the latter aspect, the questionnaire asked the mothers to report whether their children had a chronic disease or condition that required one or more regular health checks by a doctor in 2019 (used as a reference period) and whether the children had any problems getting routine health checks after March 2020. In this study, we focused on children that, at the time of the questionnaire submission, resided in Italy and were affected by a chronic disease that required at least one health check in 2019. We investigated if individual and family characteristics (maternal age, child age number of diseases, number of visits in the pre-pandemic year, and household income) were associated with having experienced healthcare access problems. We used a multivariable Poisson regression to estimate the mutually adjusted prevalence rate ratio (PRR) of experiencing a healthcare access problem for each individual and family characteristic, additionally adjusting for child sex and region of residence. Given that more than one child from the same family could be included in the analyses, we applied cluster-robust standard errors to account for correlated observations. Household income was expressed as the Equivalized Household Income Indicator (EHII), which measures the equivalized disposable household income, namely the total income of a household, after tax and deductions, divided by the number of household members converted into equalized adults (8). The EHII was categorized into tertiles using the cutoffs from the NINFEA internal distribution of the EHII. As an additional analysis, we categorized the EHII into tertiles using the Italian distribution from the “pan-European Union Statistics on Income and Living Conditions” (EUSILC) 2011 survey as a cutoff. Given the low proportion of children in the lowest EHII EUSILC tertile (4.5%), we grouped children in the lowest tertile and children in the medium tertile in this analysis.

## Results

We obtained information on 3,721 children (70.1% response rate), of whom 353 (9.5%) were affected by at least one chronic disease or condition that required a medical visit in 2019. The distribution of prevalent chronic conditions is reported in Table 1. For 130 (36.8%) children affected by at least one chronic disease requiring healthcare in 2019, the mothers reported problems with the regular health checks during the pandemic period. The results from the multivariable analysis are shown in Table 2. There was no evidence of an association between children's age and number of chronic comorbidities and having experienced problems in receiving routine health checks after March 2020. Conversely, the number of pediatric visits for chronic conditions in the pre-pandemic year was associated with having experienced healthcare access problems during the pandemic. We observed a prevalence rate ratio (PRR) for experiencing healthcare access problems equal to 1.49 [95% confidence intervals (CIs): 1.12–1.99] for children who required three or more visits in 2019 when compared to children who required one or two visits in 2019. In addition, we observed that lower family income was associated with a higher risk of experiencing healthcare access problems. Using the NINFEA internal EHII distribution cutoffs, we observed that children living in families at lower income tertiles had more chance of experiencing healthcare access problems compared to children living in families at the highest income tertile (PRR for a tertile decrease: 1.22; 95% CIs: 1.02–1.46, Table 2). Similar results were obtained when using the EUSILC Italian distribution cutoffs (PRR for low/medium EHII vs. high EHII: 1.29; 95% CIs: 0.97–1.72, data not shown). Among the 130 children that missed a routine health check, 34 (26.2%) did not receive any remote support from their doctor and 2 (1.5%) had to go to the emergency department (data not shown).

**Table 1:** Access to routine visits during the COVID-19 pandemic in the population with chronic diseases of the NINFEA birth cohort stratified by disease group.

	***N***	**Difficulties in the access to routine visits**	
		**No**	**Yes**
**Disease group**		***N*** **(%**^a^**)**	***N*** **(%**^a^**)**
Respiratory	49	29 (59.2)	20 (40.8)
Allergies	43	31 (72.1)	12 (27.9)
Neurocognitive	35	19 (54.3)	16 (45.7)
Otorhinolaryngology	32	18 (56.3)	14 (43.7)
Neurological	31	14 (45.2)	17 (54.8)
Ophthalmology	24	16 (66.7)	8 (33.3)
Gastroenteric	23	18 (78.3)	5 (21.7)
Dermatologic	19	12 (63.2)	7 (36.8)
Orthopedic	19	13 (68.4)	6 (31.6)
Nephrology/urinary	17	13 (76.5)	4 (23.5)
Endocrine	14	6 (42.9)	8 (57.1)
Syndromes (multiple organ disorders)	9	6 (66.7)	3 (33.3)
Tumors	6	5 (83.3)	1 (16.7)
Other	31	23 (71.9)	9 (28.1)
All	353	223 (73.2)	130 (36.8)

^a^Row proportion.

**Table 2:** Association of individual and family characteristics with difficulties in the access to routine visits for pediatric chronic diseases in the NINFEA birth cohort.

**Characteristics**	**Difficulties in the access to routine visits during the COVID-19 pandemic**			
	**No difficulties Mean (SD)/*****N*** **(%**^a^**)**	**Difficulties Mean (SD)/*****N*** **(%**^a^**)**	**PR**_crude_ **(95% CI)**	${\text{PR}}_{\text{adj}}^{\text{b}}$ **(95% CI)**
Maternal age (years)	43.9 (4.7)	43.7 (5.3)	1.00 (0.97–1.02)	1.00 (0.96–1.03)
Child age (years)	9.9 (2.7)	9.6 (3.1)	0.98 (0.93–1.03)	0.97 (0.92–1.03)
**Number of diseases**				
1	208 (93.2)	115 (88.4)	*Reference*	
≥2	15 (6.7)	15 (11.5)	1.40 (0.95–2.07)	1.20 (0.81–1.79)
Missing	/	/		
**Number of visits in the pre-pandemic year**				
1–3	190 (85.2)	95 (74.2)	*Reference*	
>3	33 (14.8)	33 (25.4)	1.50 (1.12–2.00)	1.49 (1.12–1.99)
Missing	/	2		
**Household income (EHII)** ^c^				
High	74 (35.9)	31 (24.8)	*Reference*	
Medium	65 (31.6)	41 (32.8)	1.31 (0.89–1.91)	1.28 (0.87–1.89)
Low	67 (32.5)	53 (42.4)	1.50 (1.05–2.14)	1.49 (1.02–2.17)
Missing	17	5		
Tertile decrease			1.22 (1.02–1.44)	1.22 (1.02–1.46)

^a^Column proportion.

^b^Prevalence ratios adjusted for maternal age, child age, child sex, region of residence, number of diseases, and number of visits in the pre-pandemic year.

^c^Cutoffs were defined according to the internal NINFEA distribution of the equivalized total disposable household income.

EHII, Equivalized Household Income Indicator.

## Discussion

Some studies have shown that the availability of and access to healthcare for children with specific chronic diseases was significantly affected during the pandemic (6). The suggested mechanisms include: (i) reduced access to emergency departments, (ii) delays in diagnosis, (iii) difficulties in accessing medication, (iv) reduced surgical activity, and (v) canceled medical visits. Our study focused on this last aspect, showing that after the start of the pandemic, in March 2020, more than one-third of the children with a chronic disease or condition had problems getting routine health checks, as reported by the mothers. This proportion was higher for children requiring more visits in the pre-pandemic period and for those living in lower-income families. It has been documented that the COVID-19 pandemic disproportionately affected population strata characterized by low SEP, with higher risks of hospitalizations and adverse health outcomes (9, 10). Furthermore, the negative effects of lockdown measures, such as reduced income or job loss, have been shown to affect the most vulnerable population, including people with a lower SEP (11). Our study suggests that the COVID-19 pandemic may have indirectly increased health inequalities also by causing healthcare access problems for children with chronic diseases living in households characterized by a low SEP. The study's strength is that the questionnaire was completed by a group of mothers who are participating in the NINFEA birth cohort, which was established in 2005 and has been followed up on for many years. Therefore, the estimates were based on a well-known underlying population and were less prone to selection bias due to impact-driven participation. Moreover, we focused on the general pediatric population and considered any chronic disease or condition instead of focusing on a specific type of patient. As a limitation, we did not assess in the questionnaire the reason for having missed a routine health check (e.g., visit postponed/canceled, restrictions imposed by the pandemic, and fear of infection). Thus, we could not test which specific mechanism contributed the most to healthcare access problems, and how different mechanisms might have interacted with family SEP. In addition, we could not evaluate if a missed routine health check, as reported by the mothers, resulted in poorer clinical outcomes for the children. However, a relevant health impact is suggested by the fact that more than one-fourth of the children that missed a visit did not receive any remote support from their doctor or had to go to the emergency department. In addition, in this study, EHII was recorded at baseline (during pregnancy) and may not precisely reflect the EHII during the pandemic. However, baseline EHII is recorded before the assessment of the outcome under study and, thus, is not affected by reverse causality that might arise from the potential impact of childhood chronic diseases on the family income (12).

As the study was nested in an ongoing mother–child cohort, further follow-up of these children will be available in the future to evaluate the long-term impact of the COVID-19 pandemic on a vulnerable population such as these children affected by chronic diseases. In conclusion, our study shows that, during the COVID-19 pandemic, a proportion of children with a chronic condition, especially those living in families with low socioeconomic positions, experienced a healthcare access problem. These results should be considered if future pandemic waves and lockdown measures occur.

## Data availability statement

The raw data supporting the conclusions of this article will be made available by the authors, without undue reservation.

## Ethics statement

The studies involving human participants were reviewed and approved by Ethical Committee of the San Giovanni Battista Hospital and CTO/CRF/Maria Adelaide Hospital of Turin (project number 45). Written informed consent to participate in this study was provided by the participants' legal guardian/next of kin.

## Author contributions

GM drafted the manuscript. GM and MP performed the data analysis. All authors designed the questionnaire, planned the analysis, discussed the results, and contributed to the final manuscript.
